# King Edward's Hospital Fund for London

**Published:** 1921-01-15

**Authors:** 


### King Edward's Hospital Fund for London.

A Meeting of the General Council of King Edward's Hospital Fund for London was held on January 10 at the offices of the Fund, 7 Walbrook. The Order, signed by His Royal Highness the Prince of Wales, President of the Fund, appointing members of the General Council, members of Committees, and officers for the year 1921 was read, and the resolutions providing for the work of the Fund for 1921, which were approved at the meeting of the President and General Council held at St. James's Palace on December 14, 1920, were formally adopted.

